# ASSOCIATION BETWEEN ELDER ABUSE AND TELOMERE LENGTH IN OLDER ADULTS WITH COGNITIVE IMPAIRMENT

**DOI:** 10.1093/geroni/igz038.1818

**Published:** 2019-11-08

**Authors:** Boye FANG, Elsie Yan, Keith Tung, Gengzhen Chen

**Affiliations:** 1 Hong Kong Polytechnic University, Hong Kong Polytechnic University, Hong Kong; 2 The Hong Kong Polytechnic University, Hong Kong, Hong Kong; 3 University of Hong Kong, Hong Kong, Hong Kong; 4 Shantou University Medical College, Shantou, China

## Abstract

Objectives: Elder abuse is a stressful event that can lead to compromised psychological and physical health consequences. This study examines the association between elder abuse and telomere length (TL), a biomarker reflecting cellular aging and disease pathogenesis. Methods: Between 2015 and 2016, 1,002 older adults (aged≥55 years) with cognitive impairment were consecutively recruited from the geriatric and neurological departments of three Grade-A hospitals in Guangdong Province of People’s Republic of China. At two-year follow-up, 958 of these participants were reassessed and 600 of them were randomly selected for this study. The outcome variable is TL (measured in blood cells using a multiplex quantitative polymerase chain reaction) and the major independent variables were current experience of overall abuse, psychological abuse, physical abuse, caregiver neglect, and previous experience of domestic abuse in the past two years. Potential confounding variables include demographic variables and increased severity of cognitive impairment, neuropsychiatric symptoms, sleep disorders, and depressive symptoms. Results: Multivariate regression models show that current experience of overall domestic abuse (t= -5.116, β= -0.376, confidence interval[CI] -20.231–-9.006), psychological abuse (t= -4.431, β= -0.231, [CI] -13.023–-5.023), physical abuse (t= -2.474, β= -0.151, CI -14.116–-1.621), and caregiver neglect were associated with shorter TL (t= -4.470, β= -0.185, CI -10.457–-4.072). Other predictors of shorter TL were previous experience of domestic abuse and increased severity of depression. Discussion: Both current and previous experiences of elder abuse are associated with shorter TL. Multidisciplinary efforts were needed to prevent and intervene elder abuse.

